# Genipin-Crosslinking Effects on Biomatrix Development for Cutaneous Wound Healing: A Concise Review

**DOI:** 10.3389/fbioe.2022.865014

**Published:** 2022-05-20

**Authors:** Dewi Utami Nike, Nur Izzah Md Fadilah, Nusaibah Sallehuddin, Ahmad Yasser Hamdi Nor Azlan, Farrah Hani Imran, Manira Maarof, Mh Busra Fauzi

**Affiliations:** ^1^ Centre for Tissue Engineering and Regenerative Medicine, Faculty of Medicine, Universiti Kebangsaan Malaysia, Kuala Lumpur, Malaysia; ^2^ Faculty of Pharmacy and Health Sciences, Universiti Kuala Lumpur Royal College of Medicine Perak, Ipoh, Malaysia; ^3^ Department of Surgery, Faculty of Medicine, Universiti Kebangsaan Malaysia, Kuala Lumpur, Malaysia

**Keywords:** antioxidants, biomatrix, genipin, skin tissue engineering, wound healing, crosslinking

## Abstract

Split skin graft (SSG), a standard gold treatment for wound healing, has numerous limitations such as lack of fresh skin to be applied, tedious process, severe scarring, and keloid formation followed by higher risks of infection. Thus, there is a gap in producing polymeric scaffolds as an alternative for wound care management. Bioscaffold is the main component in tissue engineering technology that provides porous three-dimensional (3D) microarchitecture for cells to survive. Upon skin tissue reconstruction, the 3D-porous structure ensures sufficient nutrients and gaseous diffusion and cell penetration that improves cell proliferation and vascularization for tissue regeneration. Hence, it is highly considered a promising candidate for various skin wound healing applications. To date, natural-based crosslinking agents have been extensively used to tailor the physicochemical and mechanical properties of the skin biomatrix. Genipin (GNP) is preferable to other plant-based crosslinkers due to its biological activities, such as antiinflammatory and antioxidant, which are key players to boost skin wound healing. In addition, it has shown a noncytotoxic effect and is biocompatible with human skin cells. This review validated the effects of GNP in biomatrix fabrication for skin wound healing from the last 7 years of established research articles and stipulated the biomaterial development-scale point of view. Lastly, the possible role of GNP in the skin wound healing cascade is also discussed. Through the literature output, it can be concluded that GNP has the capability to increase the stability of biomatrix and maintain the skin cells viability, which will contribute in accelerating wound healing.

## Introduction

**FIGURE 1:**
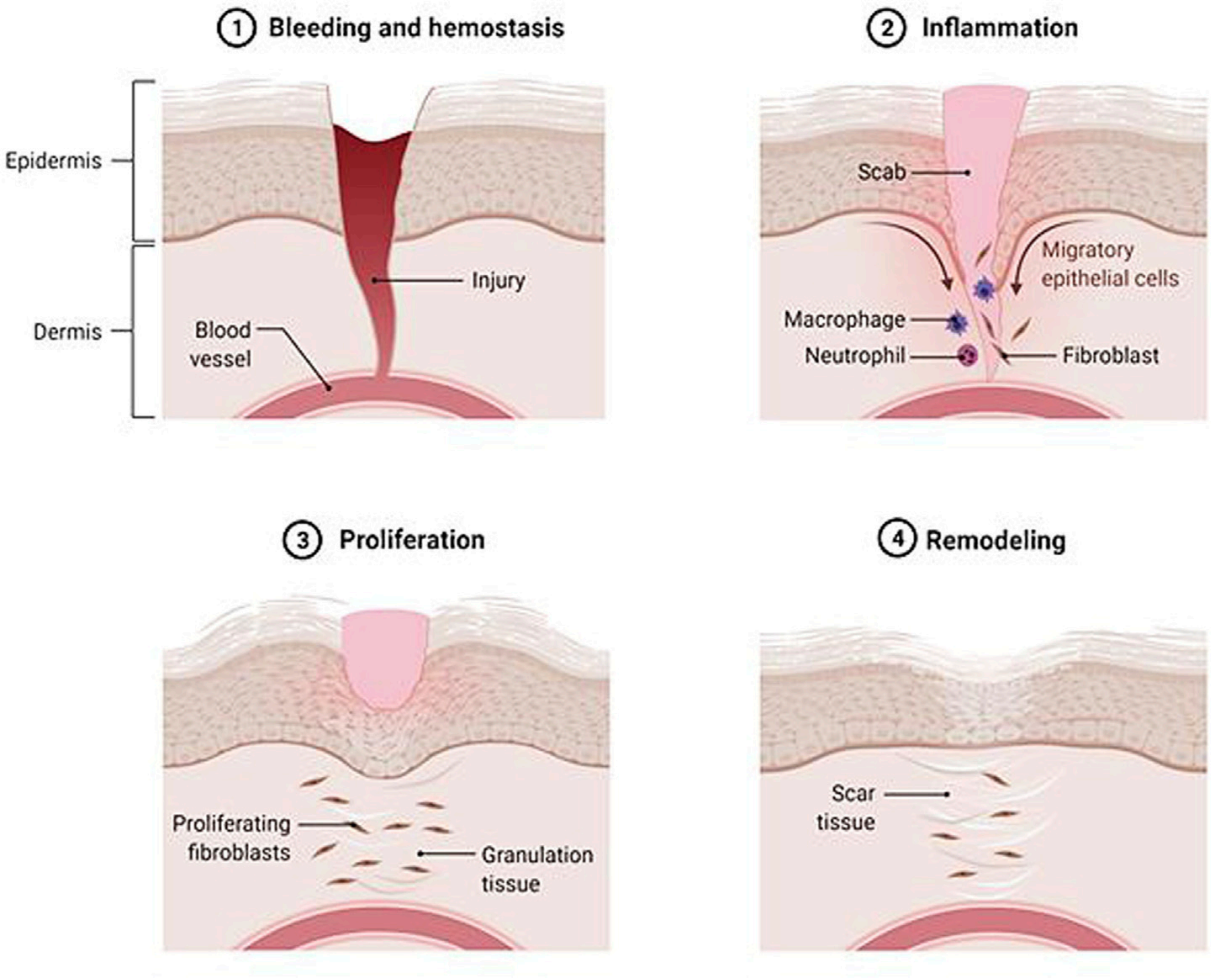
Wound healing performance. The closure of wound occurs in four overlap steps: hemostasis, inflammation, proliferation, and remodeling.

Skin is an important part of the human body that gives vital contributions through different functions, including hydration, protection from pathogens, excretion mechanism, and thermal regulation. It is composed of different layers, including epidermis, dermis, and subcutaneous tissue, which are sticky and soft. Skin wound can be interpreted as the deterioration or disturbance of normal skin functions due to chemical, physical or thermal injury. It is a leading burden on healthcare with increasing cases in the world. The Global Burden of Disease (GBD) study reported a prevalence of 605,036,000 cases in 2015 compared to 492,883,000 cases in 2005 based on global, regional, and national data from over 195 countries and territories (Utami et al., 2020; Chouhan et al., 2019; Schlottmann et al., 2021). The complete wound healing process is carried out in four main parallel steps; hemostasis, inflammation, proliferation, and remodeling (Figure 1). The first response after the creation of the wound is the activation of platelets to form a fibrin clot. The inflammation stage is mediated by macrophages and neutrophils to block bacterial invasion. During the proliferative phase, the wound bed is filled with growing skin cells and growth factors. Meanwhile, the last phase of wound healing (remodeling) consists of deposition to the extracellular matrix (ECM) and subsequent reconstitution of granulation tissue to scar tissue (Chouhan et al., 2019; Salleh and Fauzi, 2021; Yazarlu et al., 2021; Zawani and Fauzi, 2021).

**FIGURE 2:**
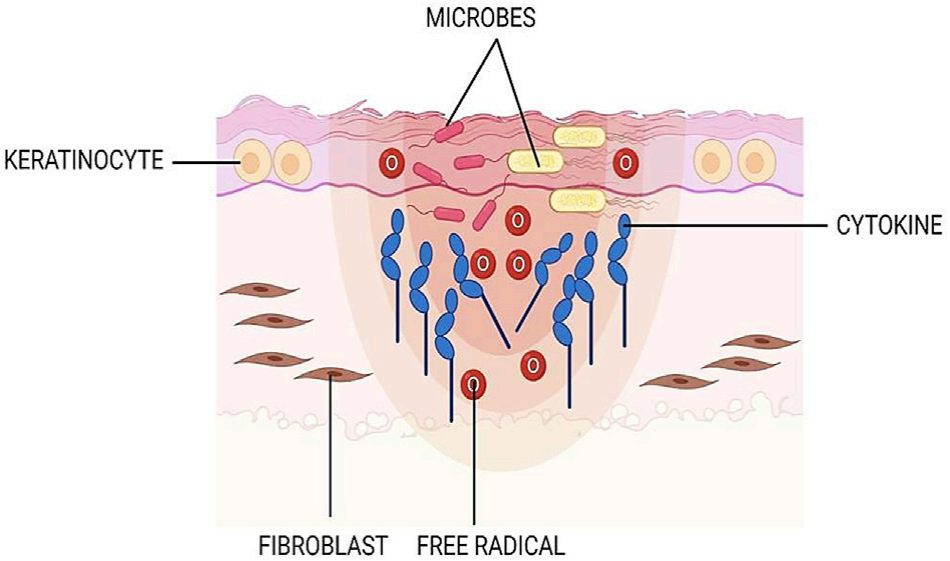
Chronic wound. In the late-healing wound, there are excessive levels of bacteria, cytokines, and free radicals.

In general, skin wounds can be categorized into acute and chronic based on the healing duration. In the United Kingdom, the annual wound case is predicted to be elevated by 9% for acute and 12% for chronic. The closure of chronic wounds takes a longer time than of the acute injury. The chronic wound has been assigned as a major public health problem associated with very high economic costs, representing 2–4% of the total health system costs in the West (Magin et al., 2016; Rodrigues et al., 2019; Azevedo et al., 2020). Maaz Arif et al.( 2021) and Kielo-Viljamaa et al. (2021) reported that approximately 1–2% of the population in the developed countries suffers from chronic wound. As has been explained by other authors, the hindered closure of nonhealing (chronic) wounds (namely pressure ulcer, static venous ulcer, and diabetic wounds, etc.) is occurred due to prolonged inflammation, microbial infection, and high level of free radicals (Figure 2) that leads to a failure in resuming the proliferative stage. Thus, multiple strategies should be used to prevent or treat chronic wounds. They should involve treatment that gives an antioxidant effect, suppresses inflammation, and supports skin cell proliferation (Gao et al., 2020; Przekora, 2020; Xu et al., 2020; Masri and Fauzi, 2021).

## Skin Biomatrix Development

Autologous split-thickness skin grafting (SSG) represents the standard gold approach to treating skin wounds despite intensive research activities. Unfortunately, the limited availability of fresh skin has become the disadvantage of this approach, mainly for large-size wounds. Additionally, it requires complicated surgical procedures and long-time post-procedure control, leading to higher treatment costs (Magin et al., 2016; Busra and Lokanathan, 2019; Chouhan et al., 2019; Rodrigues et al., 2019). Wound dressing (in the form of a bandage, gauze, cloth, hydrocolloid, etc.) is another choice in wound care management. Nevertheless, the commercially existing wound dressings, such as Tegaderm^®^, Mepitel^®^, Hydrofera Blue^®^, and SUPRATHEL^®^, were found to be insufficiently effective in enhancing wound closure and stimulating the functions of uninjured skin (Son et al., 2019).

Advanced tissue engineering technology has been established as an alternative treatment for various wound types to cover those issues. It has been developed to stimulate the wound microenvironment, while also having the therapeutic potential to repair skin tissue defects. Over the past decades, different biomaterials, such as polymers (natural or synthetic), have been considered one of the best options for skin reconstruction. The biomaterials were used to produce skin scaffolds (Gaharwar et al., 2020; Ratner and Zhang, 2020).

Various types of skin scaffolds have been fabricated, for example, hydrogel, foam, sponge, film, etc. A set of requirements should be taken in designing scaffolds including their biocompatibility, bioactivity, biodegradability, and physicochemical properties. They should be constructed to fit a suitable niche for promoting the new tissue formation, remodeling, vascularization, and integration. In addition, they should be porous, with a specific pore size that is suitable for cell migration and stable to provide both the diffusion of nutrients and metabolites. The stiffness property, which influences the scaffold’s degradation and stability during implantation, is highly addressed. Furthermore, they should not restrict the secretion of ECM or its remodeling process and need to show optimum fluid absorption ability in order to remove excess exudates. Many scaffolds have been reported to be effective and convenient to be used as wound healers. They were capable of preventing the wound from worsening and improving the rate of wound healing (Busra et al., 2017; Mir et al., 2018; Rahmati et al., 2020).

## Stability Enhancement *via* Crosslinking Techniques

The polymeric scaffold is a prominent element in skin tissue engineering that acts as a provisional biotemplate to support skin cell survival and tissue regeneration. Its three-dimensional (3D) porous formation is an ideal environment for skin cells to attach and proliferate. Therefore, it plays pivotal roles as wound dressings, tissue-engineered skin substitute (TESS), acellular skin substitute, and other wound treatments that offer speedy healing (Kailani et al., 2016; Arif et al., 2020; Selvarajah et al., 2020a; Tottoli et al., 2020). Nevertheless, some polymers will easily become collapse, soft, and fluidic and undergo fast degradation at body temperatures, such as natural-based polymers (gelatin, elastin, collagen, alginate, etc.) and thermoplastic polymers (polypropylene, polyethylene, polyvinyl chloride, polyvinyl alcohol, etc.). Additionally, several drawbacks including systemic toxicity primarily from byproducts, lack of mechanical strength, and instability (easy to degrade) in aqueous conditions suggest a restriction for long-term applications. The crosslinking approach, by using genipin (GNP) as an example, is the eminent strategy for biomatrix fabrication to improve those limitations for targeting future clinical outcomes in improving wound healing. In addition, it provides a lot of benefits to the fabricated biomatrix such as 1) improving mechanical strength and toughness, 2) reducing solubility in enzymatic exposure and liquid environment, 3) imparting thermal stability and swelling ability, and 4) enhancing elasticity and viscosity (Bardhwaj et al., 2018; Busra and Lokanathan, 2019; Son et al., 2019). Crosslinking can be defined as a mechanism of two or more molecules chemically joined in stabilizing the end-products. It will create an ionic or covalent bond that binds one polymer chain to another and further forms a network structure that is more stable and less reactive due to a lack of moving capability (Turner et al., 2017; Krishnakumar et al., 2018; Shah et al., 2019; Wong et al., 2019; Selvarajah et al., 2020b).

**FIGURE 3:**
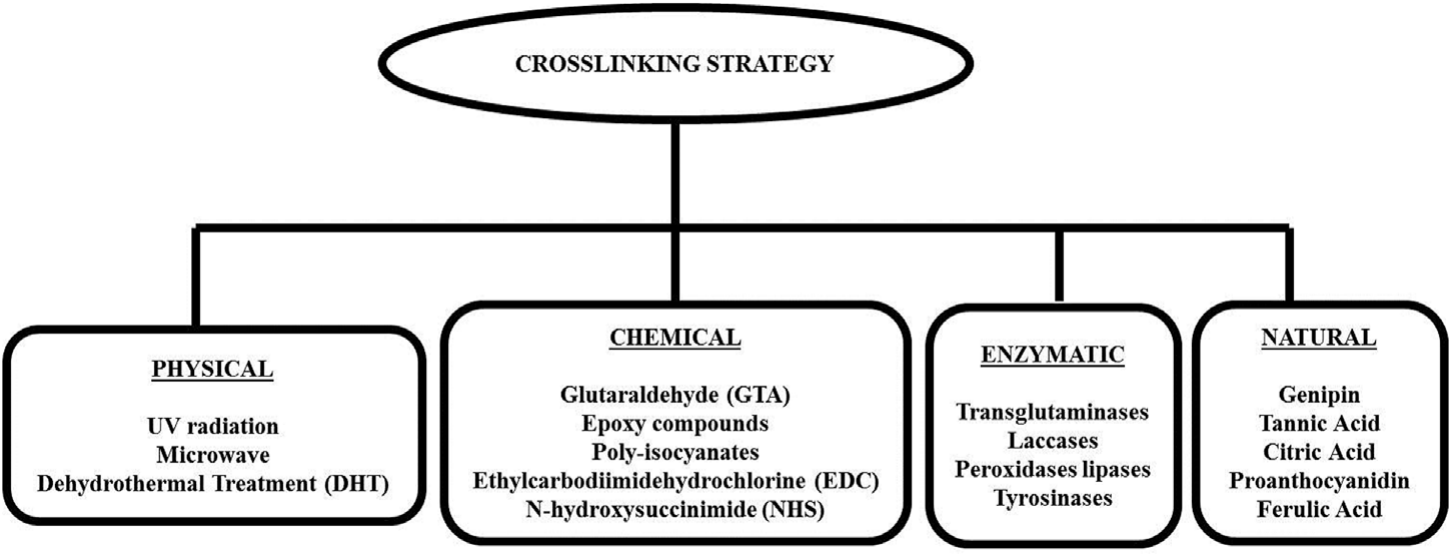
Type of crosslinking approaches. Generally, there are four categories of crosslinking methods: physical, chemical, enzymatical, and natural.

Generally, crosslinking methodologies can be varied into four types; physical, chemical, natural, and enzymatical, as shown in Figure 3. The selection of suitable crosslinking techniques is based on the type of polymers and expected properties. Physical crosslinking is a traditional method via ultraviolet (UV) radiation, microwave, and dehydrothermal treatment (DHT). However, they are no longer considered in the production of biomatrices because the aforementioned treatments produce scaffolds with less structural integrity, leading to the insufficient mechanical properties required for their applications. These methods were reported to provide a lower degree of crosslinking. In addition, UV radiation is only effective for thin and or transparent scaffolds, allowing the UV to go through the structure. The most common chemical crosslinkers including glutaraldehyde (GTA), formaldehyde, epoxy compounds, 1-ethyl-3- (3-dimethylaminopropyl) carbodiimide (EDC), and isocyanate compounds have been utilized in tissue engineering applications to achieve physicochemical and mechanical stabilization of the biomatrices. Nonetheless, the aforementioned crosslinkers exhibited a certain level of cytotoxicity due to the presence of unreacted moieties of both the crosslinkers and byproducts produced during the reactions. The main disadvantages of chemical crosslinking are the unreacted crosslinkers inside the scaffolds which ultimately elevates the formation of toxic products and limits the mechanical strength. Meanwhile, the enzymatic alteration is performed by addressing transglutaminases (TG2), laccases, peroxidases lipases, and tyrosinases as substitutes for toxic chemical approaches. The degree of crosslinking and mechanical properties are not able to be manipulated through this modification. In order to overcome those issues, natural alternatives such as GNP, tannic acid, citric acid, proanthocyanidin, and ferulic acid are preferred (Hoque et al., 2015; Reddy et al., 2015; Karaki et al., 2016; Arif et al., 2019; Wong et al., 2019; Ilkar Erdagi et al., 2020) and worth to be explored further for future use.

## Genipin as Natural-Based Crosslinking Compound

The insertion of crosslinking agents into the biomatrix formulation is essential to produce a rigid or versatile 3D bioscaffold depending on specific applications. Natural-based crosslinkers have been widely selected due to their capability to stabilize the 3D bioscaffolds, promote biocompatibility, and low toxicity effect. GNP has been well-reported to be 5,000–10,000 times less cytotoxic than other crosslinking molecules with the LD_50_ of approximately 200 mg/kg in the mice model. The survival and proliferation rate of seeded cells in GNP-crosslinked biomatrix was 5,000-fold greater than that in GTA-crosslinked biomatrix. GNP successfully suppressed inflammation and displayed an excellent biological safety profile after implantation *in vivo* studies (Manickam et al., 2014; McGann et al., 2015; Bellefeuille et al., 2017; Ubaid and Murtaza, 2018; Wang et al., 2020). Nevertheless, the selection of other related crosslinking agents, mainly natural-based crosslinkers, should depend on the intended strategies in improvising the bioscaffold fabrication Table 1.

**TABLE 1:** Comparison between GNP and other crosslinking methods.

Point No.	Type of crosslinkers	Toxic effect	Stability effect	Antiinflammation activity	Antioxidant activity
1	GNP	No	High	Yes	Yes
2	EDC	Yes	Low	No	No
3	DHT	Yes	Low	No	No
4	GTA	Yes	Low	No	No
5	TG2	Yes	Low	No	No

**FIGURE 4:**
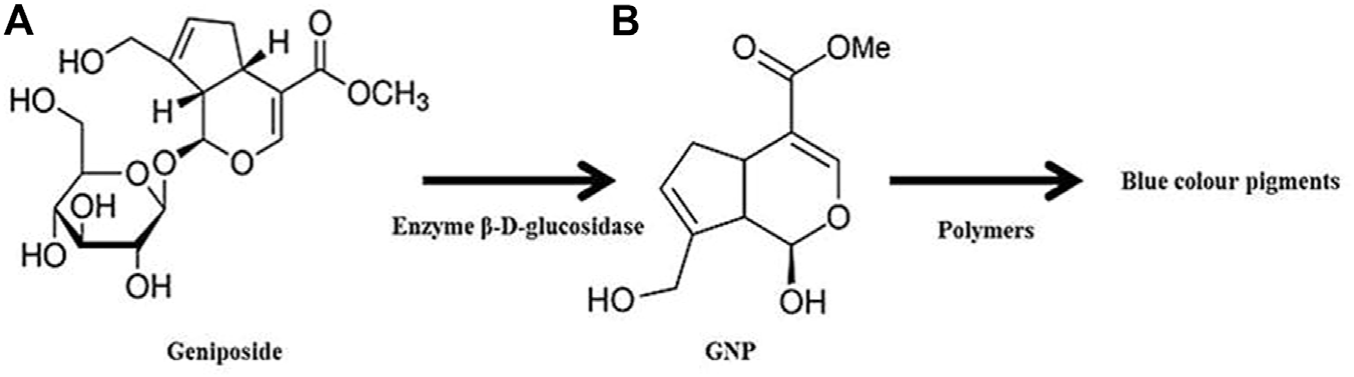
GNP production through geniposide hydrolysis. Crosslinking reaction between GNP and polymers leads to the formation of dark blue pigments.

Briefly, GNP is a colorless aglycone extracted from *Gardenia jasminoides* (*G. jasminoides*), *Gardenia fructus* (*G. fructus*), and genipap fruit [from *Genipa americana* (*G. americana*)]. *G. jasminoides*, locally named Cape jasmine, is a plant that belongs to the Rubiaceae family which grows wildly in Vietnam, Southern China, Taiwan, Japan, Myanmar, and India. It has been reported that the geniposide in ripe *G. jasminoides* was hydrolyzed into GNP through the bacterial enzyme β-d-glucosidase as demonstrated in Figure 4. *G. americana*, also known as genipapo, is a plant that grows in northern South America, the Caribbean, and southern Mexico. The fruit is called genipap. GNP is obtained from genipap extract *via* high-pressure processing based on enzyme-assisted extraction in a two-phase aqueous system. Until now, this method has resulted in the highest GNP concentration (196 mg/g). The immobilized glycosyl hydrolase family three β-glucosidase has been effectively employed to convert geniposide into GNP through hot water extraction of *G.* fructus (Son et al., 2015; Habtemariam and Lentini, 2018; Ubaid and Murtaza, 2018). In addition, GNP was also isolated from the methanolic extract of *Apodytes dimidiate*, which belongs to the family Icacinaceae, and the bark or fruit of *Rothmannia wittii*. Due to the low yield of GNP obtained from each extraction process, the market price of GNP becomes prohibitive (Shanmugam et al., 2018).

GNP, C_11_H_14_O_5,_ or methyl 1-hydroxy-7-(hydroxymethyl)-1,4a, 5,7a-tetrahydrocyclo -penta [c] pyran-4-carboxylate, comprises a dihydropyran ring and an ester group with molecular weight 226.226 g/mol and melting point at a temperature of 120–121°C. It is a white crystalline powder soluble in several solvents such as water, saline, acetone, ethyl acetate, propylene glycol, and alcohol (methanol or ethanol). Under acidic and neutral conditions, it reacts spontaneously with primary amines from the polymer chain and the end-product appears as dark blue pigments (Figure 4). The Chinese traditional medicine employed GNP to treat pyrogenic infection, febrile disease, sprain, swelling, liver apoptosis, inflammation-driven diseases, diabetes mellitus, and jaundice (Shanmugam et al., 2018; Ubaid and Murtaza, 2018). GNP will be enzymatically hydrolyzed after oral consumption by the β-glucosidase that was secreted by intestinal bacteria. Numerous researchers also highlighted its biosafety and wide-range pharmaceutical profits (Son et al., 2015; Sohn et al., 2017; Shah et al., 2019; Wang et al., 2020). As has already been explained earlier, the treatment that can reduce inflammation and oxidative stress has become the best option to force wound healing. Hence, GNP may potentially assist in wound healing progress through its antiinflammatory and antioxidant properties, which are essential contributors to the healing cascade (Utami et al., 2020).

**FIGURE 5:**
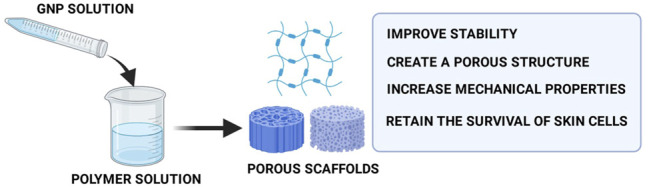
Functions of GNP in scaffold fabrication. The addition of GNP into the scaffolds will produce porous scaffolds with superior mechanical strength that causes shape-retaining scaffolds.

According to previous literatures, the crosslinking procedure was carried out by adding GNP into the polymer solution, mixing GNP solution with a polymer solution, or soaking the prepared biomatrix in GNP solution (Selvarajah et al., 2020b; Ilkar Erdagi et al., 2020; Karaki et al., 2016; Wang et al., 2020; Manickam et al., 2014; Aubert-Viard et al., 2019; Elliott et al., 2015). The pre-crosslinking mixing is a good choice for unstable noncrosslinked biomatrix because it gives the material more rigid structure and improves physical properties. To the best of our knowledge, the last method, in which the biomatrix was immersed into the GNP solution, can be selected for scaffold fabrication. The crosslinking reaction of GNP occurs in mild conditions (room temperature), which is more advantageous for highly thermo-sensitive polymers. GNP has been used to manipulate various skin-based biomatrices, which are produced from gelatin (GEL), chitosan (CH), collagen (COL), polyethylene glycol (PEG), hyaluronic acid (HA), fibrin (FIB), agarose (AGAR), and many more due to their natural crosslinking ability. Numerous researchers have confirmed the formation of porous structures inside the bioscaffolds postmodification with GNP. Furthermore, GNP can form a permanent crosslink between two polymers through intra- and inter-molecular crosslinking reactions, producing scaffolds with higher biomechanical properties in the concentration range of 0.1–0.5% (Turner et al., 2017; Krishnakumar et al., 2018; Aubert-Viard et al., 2019; Sulistiawati Rusyanti et al., 2019; Elliott et al., 2015; Hobbs et al., 2018). The porous microstructure and higher modulus and mechanical strength will lead to an acceptable *in vitro* biocompatibility (Figure 5) (Cabiati et al., 2018; Gattazzo et al., 2018; Nyambat et al., 2020). Moreover, it ensures adequate nutrient and oxygen diffusion during skin regeneration as well as cell penetration to support cell proliferation and vascularization. As already established, the proliferation of cells is vital in wound-healing performance. Some experiments demonstrated that 0.22–1 mM of GNP was not toxic and able to retain the viability of skin cells. Moreover, the GNP-crosslinked scaffolds were characterized by a broad molecular weight range and resistance toward biodegradation which could be useful for skin regeneration as faster degradation may cause the loss of scaffolds ability prior to forming a new matrix structure. Too slow or quick degradation can promote fibrosis and/or insufficient support for tissue growth (Fauzi et al., 2019; Arif et al., 2020).

In addition, GNP reactivity depends on its concentration, crosslinking temperature, duration, and pH key factors, which are reduced at low pH, allowing control over crosslink density. In addition, it tends to undergo spontaneous polymerization at basic pH. The stiffness changes due to modification in crosslink density by GNP concentration can be quantified *via* coloration changes, primary amine content, and fluorescence (Elliott et al., 2015; Turner et al., 2017; Hobbs et al., 2018). The crosslinking timing is another important indicator to ensure a reasonable trade-off between the fabrication time and shape retention (Montemurro et al., 2017). In this review, the functions of GNP as a crosslinking compound in the production of biomatrix for skin wound healing were explored and summarized. The detailed mechanism of crosslinking reaction was also further explained. In addition, the possible wound-closure activities of GNP were further discussed.

**FIGURE 6:**
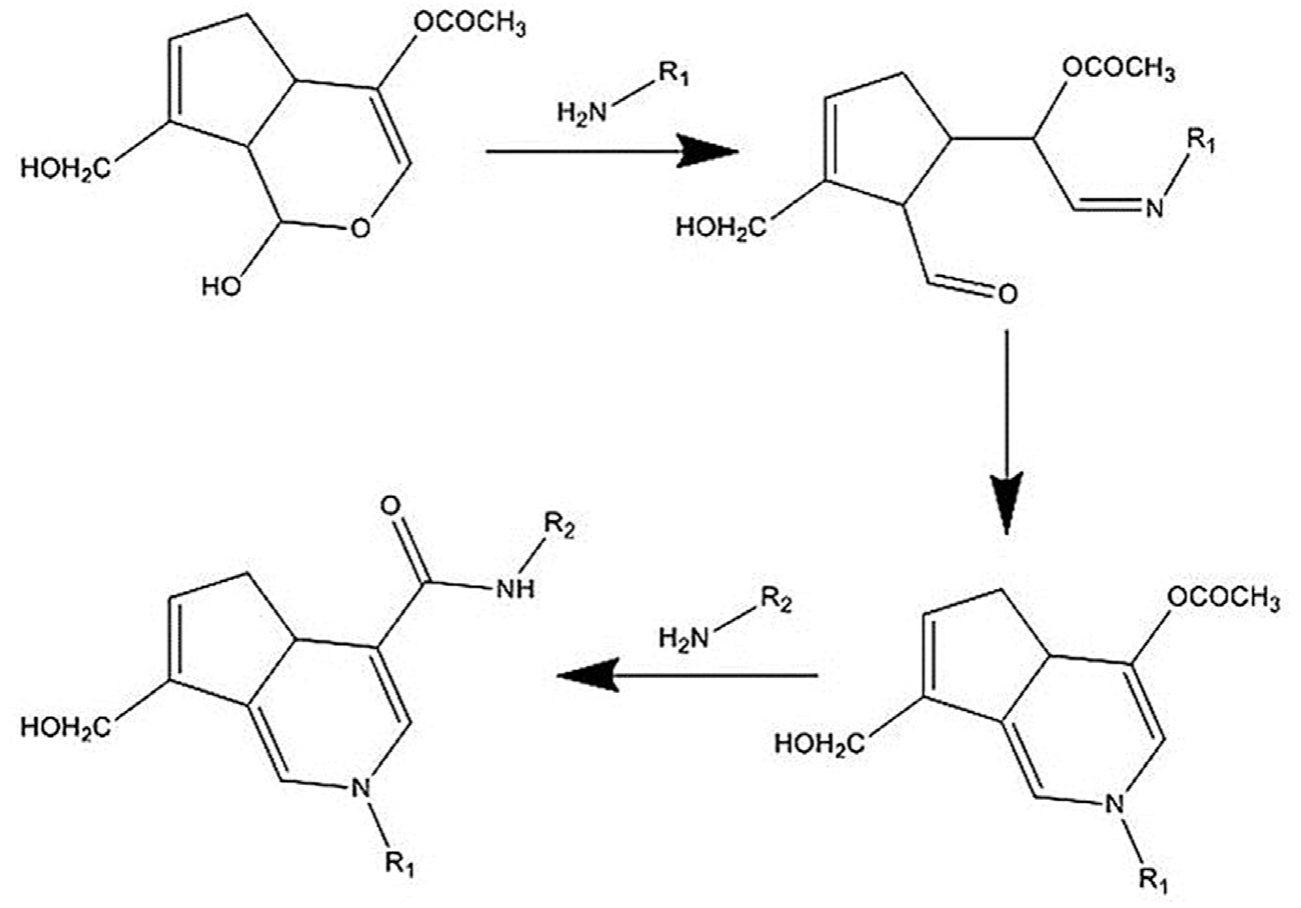
Mechanism of the crosslinking reaction. GNP will bind to the amine groups from polymer chains and then act as a polymer connector. The connection bridge between two polymer chains will lead to stability improvement.

The mechanism of the GNP crosslinking reaction is still unknown. With this in mind, many researchers proposed that GNP crosslinking process happened through two primary amine groups of polymers, involving several pathways and intermediates, to produce a dimeric product, which then promotes blue color to the solution. In general, the mechanism of the reaction is divided into two subsequent steps. The initial step is nucleophilic attack of an amine group in polymer chain to α,β-insaturated ester in GNP molecule, leading to an open ring of GNP. Next, another amine group from the polymer chain will attack the methoxycarbonyl group to produce a secondary amide-type linkage, with methanol release, to form the crosslinked compound (Wang et al., 2020). Figure 6 below shows the possible reaction that occurred upon GNP crosslinking.

Ilkar Erdagi et al. (2020) have suggested that the crosslinking reaction between GEL and GNP occurred through two different pathways under mild acidic or neutral conditions. The first reaction is a nucleophilic attack of the amino groups in the GEL ring to the oleofinic carbon atom (C3) the in GNP molecule, followed by the opening of the carbonyl and dihydropyran ring. Secondly, a nucleophilic attack of the amino groups in GEL to the carboxyl group of GNP leads to an amide formation. The further reaction involves an oxygen radical-induced polymerization of GNP that might be generated among GNP molecules that have already crosslinked with amino groups of GEL. This might lead to GNP copolymers that possess high conjugation of C=C. Plus, it is possible to be responsible for the bluish color of the GEL scaffolds.

Moreover, Muzzarelli et al. (2015) and Liu et al. (2019) have proposed the reaction mechanism between CH and GNP. The crosslinking reaction mechanism is different and occurs spontaneously in an acidic and neutral environment. The amino group in the CH ring attacks the olefinic carbon atom C-3 in the GNP ring and is followed by the opening of the dihydropyran ring. After that, the opening ring is attacked by the secondary amino group on the newly formed aldehyde group. In neutral pH, the GNP ring-opening reaction occurs through nucleophilic attack by hydroxyl ions in an aqueous solution to form intermediate aldehyde groups, which subsequently undergo aldol condensation. Finally, the terminal aldehyde group on the polymerized GNP undergoes a Schiff reaction with the amino groups in the CH ring to form crosslinked networks. The crosslinking effects could be different depending on the materials involved and may affect the physicochemical and mechanical strength followed by the cellular interaction.

## Genipin Crosslinking Effect on Biomatrix Properties

### Swelling Property

Swelling behavior is an essential parameter of the skin biomatrix for wound care. It reflects the ability of scaffolds to absorb exudates and toxins at the wound site. The water molecule will bind into the polymer chains and create a cage-like network. The excess water will then enter freely into the 3D-scaffold network and result in swelling without being dissolved. Therefore, a biomatrix must have a higher fluid uptake capability for wound applications with high exudates. Some previous studies have revealed the ability of GNP to maintain the shape fidelity of scaffolds in an aqueous environment (Habtemariam and Lentini, 2018; Rodriguez-Rodriguez et al., 2019). Therefore, the presence of GNP is played a vital role in the development of skin scaffolds. The swelling studies were commonly conducted using phosphate buffer saline (PBS) solution or water to mimic the exudates in the wound site.

COL, CH, and HA have been employed as biomaterials by Lewandowska-Lancucka et al. (2019) to prepare hybrid injectable hydrogels. They loaded the systems with various ratios of amino-functionalized silica particles and modified them with GNP. The swelling ability was investigated by incubating the hydrogels in PBS solution for 24 h at a temperature of 37°C with gentle shaking. After 24 h, they found that all hydrogel groups exhibited a high swelling ratio (>1,000%). Hafezi et al. (2019) have developed 3D printed GNP-crosslinked CH films. In order to evaluate the swelling index, the films were immersed for 120 min in PBS solution (pH 7.4 ± 1) at a temperature of 37 ± 1°C. The optimum swelling behavior of CH-GNP film was 940 ± 99%. PEG600 was added to the CH-GNP films for further investigation and the swelling capacity was found to increase gradually to 985 ± 165%. Both CH-GNP and CH-GNP-PEG600 films swelled rapidly within the first 5 min and began to be unstable in PBS within 30 min. A steady-state was reached within 80 and 90 min for CH-GNP and CH-GNP-PEG600, respectively.

Liu et al. (2016) have experimented with fabricating films from CH, PEG, titanium dioxide (TiO_2_), and silver (Ag). They altered the films *via* GNP crosslinking. The swelling of GNP-CH, GNP-CH-PEG, GNP-CH-PEG-TiO_2,_ and GNP-CH-PEG-TiO_2_-Ag was validated by soaking them in a buffer solution with various pH. The results demonstrated that all films offer a high swelling ratio (>200%). Furthermore, the swelling of the GNP-CH-PEG matrix was pH-dependent, while GNP-CH-PEG-TiO_2_-Ag nanocomposites swelled enormously in the acidic medium compared to the neutral or basic one. COL and CH-based scaffolds were manufactured and further manipulated by using GNP, by Perez-Puyana et al. (2020). They prepared the scaffolds with two different biopolymer concentrations (1 and 2 wt%). The measurement of the swelling ratio was conducted by using water as a medium at 25 ± 1°C and pH 7.0 ± 0.4. The investigation exhibited a remarkable swelling ratio for all formulations and an increasing trend of swelling ratio, in the 1 wt% formulation, with the addition of GNP at a higher concentration (except for the hybrid system, due to the synergy that was produced between both polymers). In contrast, the 2 wt% groups showed a decreasing swelling ratio trend when a higher GNP concentration was incorporated. This phenomenon occurred probably due to more polymer chains in the structure, favoring the formation of crosslinking intramolecular bonds, obtaining less porous structures, and therefore, a lower swelling capacity.

In a research performed by (Ilkar Erdagi et al., 2020), GNP-crosslinked GEL-diosgenin-nanocellulose (DG-NC) hydrogels were submerged in PBS solution with different initial pH (4.5, 5.5, 6.5, 7.5, 8.5, and 9.5) at a temperature of 37°C for 24 h to reach equilibrium swelling. The crosslinked hydrogel systems were reported to have a good water uptake capacity (>200%) and reached maximum swelling in an acidic environment (pH 4.5) due to the protonation of free primary amino groups on the GEL backbone. Roether et al. (2019) determined the swelling capacity of hyaluronic acid (HA)-based cryogel, which was crosslinked with GNP (HA-GNP) cryogel, by soaking the cryogel in the water. The reaction of HA and GNP was found to form a stable intermolecular bond that was strong enough to allow the construct to swell in water without being dissolved. The cryogel was reported to exhibit long-term stability for several months, which is considered an important characteristic for wound healing applications. The study described that no stable structures could be obtained without GNP intervention. Fauzi et al. (2019) did a swelling comparison observation of OTC-I sponges with the addition of GNP and EDC. They reported a lower swelling ability of EDC-crosslinked formulation than GNP-crosslinked formulation. A year later, they determined the swelling of OTC-I sponges in the presence of GNP and EDC. The outputs demonstrated similar findings to their previous research; higher swelling in the GNP-crosslinked group (1886 ± 56%) than EDC-crosslinked group (1,658 ± 62%) (Fauzi et al., 2020). The swelling capacity of GNP-crosslinked CH-alginate (ALG)-HA polyelectrolyte composite sponges was monitored by Meng et al. (2021). They saw high swelling for all formulations after 2 h of the experiment (by using low field NMR). A 100% wound closure was achieved in the *in vivo* study on the dorsum of Wistar rats. The accumulated data proves that GNP exposure was able to stabilize skin biomatrix in aqueous conditions.

### Enzymatic Biodegradation

Enzymatic biodegradation is the physical deterioration of the scaffolds after being exposed to an enzyme. Stable integrity of the scaffolds in biological pH and ion is crucial to maintain mechanical property and porosity for an excellent cellular response. Furthermore, as mentioned earlier, the integrity of scaffolds can be enhanced *via* crosslinking modification. Thus, the addition of GNP into the scaffolds formulation for skin wound healing is highly recommended. Enzyme solutions, such as collagenase, lysozyme, and trypsin, were commonly used in biodegradation tests to mimic the *in vivo* microenvironment of matrix degradation, resulting in gradual dissolution into the supernatant. The degradation process occured due to the cleavage of intermolecular bridge between GNP and polymers by enzyme solution (Manickam et al., 2014; Raja and Fathima, 2018; Fang et al., 2020).

Raja and Fathima (2018) have developed a GNP-crosslinked GEL hydrogel composite containing an optimized concentration of cerium oxide nanoparticles (G-Onp) for wound care application. A trypsin solution (0.1 mg/ml) was added to the sponge biomatrix to evaluate the degradation rate. They hypothesized that G-Onp had shown decreased degradation rate (%) compared to the noncrosslinked GEL. More than 98% of GEL was utterly degraded within 2 h, while G-Onp lasted until 3 h. Upon 12 days of *in vivo* experiment, they found that G-Onp-treated rat has shown more prominent collagen deposition than GEL and noncrosslinked groups. In another study, the biodegradability of GNP-crosslinked CH-GEL scaffold was studied through lysozyme degradation research by Fang et al. (2020). The degradation rate for the composite scaffolds in PBS solution without lysozyme was in the range of 15–20% after a week, 45–55% after 2 weeks, 50–60% after 3 weeks, and 60–70% after 4 weeks. A fast degradation has been demonstrated in lysozyme solution due to the cleavage of glycosidic linkage between the monomers by lysozyme. However, the GNP-crosslinked CH-GEL scaffold remained after 28 days in lysozyme solution. The *in vivo* wound healing was observed in the animal wound infection model, and it was shown that the GNP-crosslinked CH-GEL scaffold improved wound closure, reepthelialization, and collagen deposition.

Turner et al. (2017) (Manickam et al., 2014) developed GEL microspheres which were then crosslinked with GNP. The crosslinked GEL microspheres were exposed to collagenase II solution to characterize their proteolytic degradation rate. The results indicated that the crosslinked scaffolds were degraded completely over 20 h in 5 U/mL collagenase and over 10 h in 25 U/mL collagenase. Another degradation test in the collagenase-enriched human-stimulated medium was performed by Valsceanu et al., 2020 to verify *in vitro* stability of graphene oxide (GO) reinforced CH-GEL film crosslinked with GNP (CH-GEL-GNP-GO). After 90 days of enzymatic exposure, the uncrosslinked formulation was fully degraded while crosslinked groups were still able to maintain their shape. In a short duration of collagenase exposure, CH-GEL-GO composition exhibited superior stability to CH-GEL. However, CH-GEL-GO has shown more inferior stability than CH-GEL-GNP-GO. The crosslinked formulation seems to be the most stable scaffold in the enzymatic environment. These findings suggested that GNP crosslinking could efficiently improve the stability of wound dressing in enzymatic conditions, extended the usage period, and reduced the frequency of wound dressing changes. Selvarajah et al. (2020b) have determined the *in vitro* biodegradation of GNP-crosslinked GEL sponge in collagenase type I solution. The study figured out that GEL sponges (7% and 10%) crosslinking with GNP 0.5% remained undissolved after 3 days of collagenase exposure and fully degraded at day 5. In addition, they suggested that the degradation of GEL sponges could be delayed by increasing the concentration of GNP. Another study performed by Annalamai et al. (2018) exhibited that the GNP-crosslinked GEL microsphere was steady and sustained over 2 days in 5.0 mg/ml collagenase solution. Meanwhile, the degradation of the scaffolds was negligible in lower collagenase concentration (1.0 mg/ml). Aubert-Viard et al. (2019) confirmed the stability of PET-CH dressing crosslinked with GNP after 5 weeks of incubation in PBS solution. They found that nonmodified PET-CH revealed quick degradation after 3 days of buffer exposure. Several concentrations of GNP (0.5%, 1.0%, and 2.5%) were also added, by Merk et al. (2021), into polycaprolacton/GEL formulation to produce sponges for regenerative purposes. A remaining mass of >92% was found in all GNP-crosslinked samples after 8 weeks of incubation in buffer solution. Meanwhile, only 41% of the mass was seen on the formulation without GNP. Fibroblasts could proliferate very well on the sponges for up to 23 days. All of the evidence supported that GNP controls the degradation rate and the resulting scaffolds could resist enzymatic degradation. Therefore, the degradation duration might be affected by GNP concentration followed by the type of biodegradation used in the aforementioned studies.

### Microstructure and Pore Size

As mentioned previously, tissue engineering aims to repair and regenerate damaged skin by constructing scaffolds that closely mimic the anatomy and physiology of the native skin. Thus, it is essential to achieve a similar architecture and interconnecting pores while fabricating scaffolds for skin regeneration. The microstructure of cutaneous scaffolds significantly affects the regenerated skin’s function and the activities of seeded cells. The scaffolds must be produced in 3D porous architecture and high pore interconnectivity to accelerate wound healing (Perez-Puyana et al., 2020; Merk et al., 2021). The porous structure of the scaffolds plays a major role in oxygen and nutrient diffusion, which is essential for cell adhesion and proliferation. Overall, 5 µm pore size could be suitable for neovascularization, 5–15 µm for fibroblast ingrowth, and 20–300 µm for cell penetration, and tissue regeneration. The increasing pore size provides a beneficial environment for cell penetration (Wang et al., 2017; Liu et al., 2019). The structure and pore size of skin scaffolds can be manipulated through GNP crosslinking. They are commonly examined *via* scanning electron microscope (SEM) (Ubaid and Murtaza, 2018; Yao et al., 2019; Masri and Fauzi, 2021).

Single-layer spongy CH-*Bletilla striata* polysaccharide (BSP) scaffolds were produced by Ding et al. (2017) with different BSP concentrations. The crosslinked scaffolds were fabricated by mixing CH solution, BSP solution, and GNP solution. SEM micrographs revealed the average pore size of the resulted scaffolds was within the range of 161 ± 44 μm to 201 ± 84 μm, which was acceptable for wound applications. The scaffolds were then applied to mice with full-thickness wounds. The wound observation presented that CH-GNP scaffolds promoted faster and more promising healing effects than noncrosslinked groups on day 7 after application. Yao et al. (2019) have loaded the GNP-crosslinked CH (GNP-CH) scaffolds with stromal cell-derived factor-1 (SDF-1) in PBS solution. The GNP-CH scaffolds were made through lyophilization of CH and GNP mixture solution. SEM images have shown a porous structure on the surface of 0.2%, 0.4%, and 0.6% GNP-CH scaffolds. The mean pore size for the cross-section images of 0.2% and 0.4% wt GNP-CH scaffolds were between 100 and 200 µm. In addition, the pore size of 0.6% Wt GNP-CH scaffold was found to be within the range of 200–300 µm. They further conducted *in vivo* study by using a rat model of full-thickness wound. After 14 days of GNP-CS treatment, the recovery rates were observed more than 80%. Fauzi et al. (2019) constructed ovine tendon collagen type I (OTC-I) scaffolds. The freeze-dried scaffolds were immersed in GNP and EDC solution at room temperature for crosslinking purposes. SEM photographs demonstrated that GNP-crosslinked OTC-I exhibited a higher percentage of pore size within the range of 1–100 µm and a lower percentage of pore size within 100–200 µm than EDC-crosslinked OTC-I. In addition, GNP-crosslinked OTC-I showed a higher percentage of pore size in the range of 100–200 µm compared to non-crosslinked OTC-I. Histological evaluation in animal tests demonstrated the formation of bilayer skin containing matured epidermal and dermal structure, after the 13th day, in the mice treated with GNP-crosslinked OTC-I sponge. Elliott et al. (2015) have added GNP into silk fibroin solution prior to crosslinked-hydrogel formation. According to SEM micrographs and confocal images presented in their article, GNP-modified silk hydrogel exhibited porous microstructure with increased pore size and connectivity compared to control. Confocal images supported SEM findings by showing uniform pore size within the saturated and wet hydrogel. No changes were seen, along with the depth of the gel representing homogenous gel formation. Another SEM photo of CH-GNP scaffolds, which were manufactured by drying the mixture of CH and GNP solution in the freeze dryer, was published by Dimida et al. (2017). They reported a highly interconnected porous structure with pore size within the range of 150–200 nm for the constructed scaffolds. Last year, Li et al. (2021) mixed GNP with CH solution and exosome solution to make hydrogel. They published an SEM image of exosome-loaded carboxymethyl CH hydrogel that was crosslinked by GNP. A porous structure was seen with pore size within the range of 100–200 µm. They investigated the wound healing ability in rats with cutaneous wounds and found clear skin appendages after 14 days of treatment in the hydrogel-treated rats. The accumulated results indicated that GNP crosslinking is critical in fabricating porous skin scaffolds for promoting wound healing.

### Mechanical Characteristics

Mechanical properties were investigated to evaluate the scaffold’s capability to withstand tensions during and after the implantation. An appropriate mechanical strength is needed to avoid the breakdown of skin scaffolds when the wound contracts (Busra and Lokanathan, 2019; Vlasceanu et al., 2020). In addition, the enhanced mechanical properties would further stimulate cell adhesion and proliferation in the modified scaffolds (Dimida et al., 2017; Rodriguez-Rodriguez et al., 2019). Biomechanical skin indicators change during ageing. The skin of older people becomes less tense, thinner, stiffer, and less flexible. Thus, the mechanical criteria of desired products will be varied. GNP treatment has been reported to manipulate the mechanical strength of polymer-based scaffolds. Hence, this strategy is essential in skin biomatrix fabrication for wound healing (Mahnama et al., 2016; Ceylan, 2021a; Ceylan, 2021b).

A mechanical test was done by Campos et al. (2017) in a Haake MARS III rheometer to measure G” (elastic modulus) and G” (viscous modulus) of GNP-crosslinked uncompressed and nanostructured FIB-AGAR hydrogels (FAH and NAFH). Briefly, the analysis revealed that G” values of GNP7.5%-FAH (470 Pa) and GNP7.5%-NAFH (7,000 Pa) were significantly higher than those of the noncrosslinked group. The G” values of GNP-FAH rose from 8.9 Pa to 124 Pa with ascending GNP concentration (0.1%–0.5%) and significantly higher than the noncrosslinked group. A live/dead assay kit was utilized to detect the viability of dermal fibroblasts on the top of GNP-FAH scaffolds. The fluorescence photos did not display any dead cells and proved cell viability within 48 h post-seeding. In 2018, Luo et al.(2018) have presented the tensile strength values of COL-based sponges (CS). They utilized microfibrillated cellulose (MFC) and GNP as crosslinking agents. Their findings have shown higher tensile strength of CS/2.5%MFC/0.3%GNP (605.00 ± 25.41 KPa) than CS/2.5%MFC (284.80 ± 19.88 KPa). In addition, Liu et al. (2019) have validated the mechanical strength of CH-nanosilica composite films manipulated through GNP and GTA addition. The characterization was carried out by using a TA-XT texture analyzer. The tensile strength of crosslinked films significantly increased with the maximum value of 70.31 MPa that belonged to GNP-crosslinked. GNP also had better improvement in Young’s modulus than GTA. Young’s modulus increased significantly from 413.21 MPa (uncrosslinked film) to 605.94 MPa (GNP-crosslinked film). The higher mechanical strength of GNP-manipulated film was achieved due to the hydrogen bond between GNP and CH-nanosilica.

Arif et al. (2020) explored GNP-GEL sponges’ toughness by using Instron 8,874 Tabletop Axial-Torsion Systems in dry conditions. Without the addition of GNP, the sponge exhibited low stiffness (0.08 ± 0.01 GPa). Meanwhile, the presence of 0.1% and 0.5% GNP lead to higher stiffness with the value of (0.10 ± 0.04 GPa) and (0.10 ± 0.02 GPa), respectively. They also conducted a comparison experiment versus another crosslinking method by using dehydrothermal (DHT). The finding showed that 0.5% GNP-crosslinked formulation offered higher tensile strain value (18.35 ± 0.73%) than DHT treatment (13.88 ± 1.65%). Gonzalez-Quevedo et al. (2020) have evaluated the mechanical property of nanostructured fibrin-agarose hydrogel (NAFH), that was modified with GNP (GNP-NAFH), by using a universal testing machine. The GNP-NAFH demonstrated higher mean values of Young’s Modulus and lowered stress at fracture in GNP-NAFH than NAFH. Concerning GNP-NAFH exhibited a lower value of strain fracture than NAFH that represents its higher deforming capability. To reveal the strengthening effect of GNP, Liang et al. (2019) have conducted uniaxial tensile tests to characterize the mechanical property of montmorillonite (MMT)-CH-GNP-sodium hydroxide (NaOH) and compared it with noncrosslinked MMT-CH films. The stress-strain curves displayed high strength and toughness of MMT-CH-GNP-NaOH. The tensile strength and toughness of the MMT-CH-GNP-NaOH group (226.3 MPa and 5.1 MJ/m^3^) are higher than those of the MMT-CH group (141.3 MPa and 1.7 MJ/m3). The tensile strength of MMT-CH-GNP-OH is elevated by 255% compared with the CH component (88.6 MPa). This phenomenon occurs because the terminal aldehyde groups on the polymerized GNP undergo a Schiff reaction with amino groups in the CH ring to form crosslinked networks that strengthen the MMT-CH-GNP-NaOH composites.

On the other hand, Nair et al. (2020) constructed COL-based films crosslinked with EDC, GNP, and TG2. A housefield tensile tester was applied to determine the tensile strength of the films. The data demonstrated a significant increase in the tensile modulus upon crosslinking with EDC/N-Hydroxy Succinimide (NHS) and GNP by up to 2000% and 400%, respectively, at the highest concentration. GNP provides a higher stiffness than EDC-NHS. Furthermore, EDC-NHS and GNP-treated films demonstrated higher tensile stress (up to 400% and 130%, respectively) than noncrosslinked groups. However, TG2-modified films exhibited no significant improvement in mechanical strength at low crosslinking concentration. Another comparison study of GNP and EDC crosslinking has been performed by Meng et al. (2018). They produced 3D skin equivalents from human skin cells and COL gel. Mechanical characterization, by using the Instron Series IX automated aaterials testing system (Zwick/Roell Z020), was conducted. They presented a bar graph that showed higher stiffness of GNP-crosslinked groups (>25 KPa) than the EDC-crosslinked group (<25 KPa). Recently, Ceylan et al. (2021) reported the mechanical properties (Young modulus, elongation at break, and strength) of GNP-crosslinked polyvinyl alcohol (PVA)-based membranes The data showed that their mechanical properties were acceptable to be used as skin tissue engineered scaffolds. Cytocompatibility testing, *via* MTT assay, revealed 50–80% viability of fibroblasts on the membranes. The findings mentioned earlier represented the crucial role of GNP crosslinking in producing scaffolds with appropriate mechanical values for skin regeneration. Table 2 presented the properties comparison of GNP and other crosslinkers.

**TABLE 2:** Comparison of properties between GNP-crosslinked with other crossslinkers—crosslinked and uncrosslinked groups.

Point No.	Property	Reference	Material	Outcome		
				GNP	Other crosslinking strategy	No crosslinker
1	Swelling	Lewandowska-Lancucka et al. (2019)	COL, CH, and HA	High swelling ratio (>1,000%)	NA	NA
		Hafezi et al. (2019)	CH and PEG	High swelling ratio (940 ± 99%)	NA	NA
		Liu et al. (2016)	CH, PEG, TiO_2,_ and Ag	High swelling ratio (>200%)	NA	NA
		Perez Puyana et al. (2020)	COL and CH	Remarkable swelling ratio	NA	NA
		Ilkar Erdagi et al. (2020)	GEL, DG, and NC	Good water uptake capacity (>200%)	NA	NA
		Roether et al. (2020)	HA	Stable structure	NA	Non-stable structure
		Fauzi et al. (2019)	OTC-I	Higher swelling ratio	Lower swelling ratio	Lower swelling ratio
		Fauzi et al. (2020)	OTC-I	Higher swelling ratio (1886 ± 56%)	Lower swelling ratio (1,658 ± 62%)	Lower swelling ratio (˂1,500%)
2	Degradation	Raja & Fathima (2018)	GEL and Onp	Remained until 3 h	NA	98% degraded within 2 h
		Fang et al. (2020)	GEL	Remained until 4 weeks	NA	Fast degradation
		Turner et al. (2017)	GEL	Degraded completely over 20 h	NA	NA
		Valsceanu et al. (2020)	CH, GEL and GO	Maintain the shape for 3 months	NA	Fully degraded in 3 months
		Selvarajah et al. (2020)	GEL	Remained undissolved within 3 days	NA	NA
		Annalamai et al. (2018)	GEL	Sustained over 2 days	NA	NA
		Aubert-Viard et al. (2019)	PET and CH	Stable throughout 5 weeks	NA	Degrade in 3 days
		Merk et al. (2021)	Polycaprolacton and GEL	Remaining mass >92% within 8 weeks	NA	Remaining mass 40% within 8 weeks
3	Microstructure	Ding et al. (2017)	CH and BSP	Porous structure	NA	NA
		Yao et al. (2019)	CH and SDF-1	Porous structure	NA	NA
		Elliott et al. (2015)	Silk	Porous structure	NA	NA
		Dimida et al. (2017)	CH	Porous structure	NA	NA
		Li et al. (2021)	Carboxymethyl CH	Porous structure	NA	NA
		Fauzi et al. (2019)	OTC-I	Porous structure	Porous structure	Porous structure
4	Mechanical strength	Campos et al. (2017)	FIB and AGAR	Higher elastic modulus and viscous modulus	NA	Lower elastic modulus and viscous modulus
		Luo et al. (2018)	COL	Higher tensile strength (605.00 ± 25.41 KPa)	Lower tensile strength (284.80 ± 19.88 KPa)	NA
		Liu et al. (2019)	CH and nanosilica	Higher tensile strength and Young’s modulus	Lower tensile strength and Young’s modulus	NA
		Arif et al. (2020)	GEL	Higher tensile strain (18.35 ± 0.73%)	Lower tensile strain (13.88 ± 1.65%)	Low stiffness (0.08 ± 0.01 GPa)
		Gonzalez-Quevedo et al. (2020)	FIB and AGAR	Higher Young’s modulus and lower strain fracture	NA	Lower Young’s modulus and higher strain fracture
		Liang et al. (2019)	Montmorillonite and CH	Higher tensile strength and toughness (226.3 MPa and 5.1 MJ/m^3^)	NA	Lower tensile strength and toughness (141.3 MPa and 1.7 MJ/m3)
		Nair et al. (2020)	COL	Significant increase in tensile modulus up to 400%	No significant tensile modulus improvement	NA
		Meng et al. (2018)	Human skin cells and COL	Higher stiffness (>25 KPa)	Lower stiffness (˂25 KPa)	NA
		Ceylan et al. (2021)	PVA	Acceptable mechanical strength	NA	NA
5	Compatibility	Arif et al. (2020)	COL	No toxic effect	Toxic effect	NA
		Nair et al. (2020)	COL	Pro-proliferative effect	No significant proliferation activity	NA
		Fauzi et al. (2019), (2020)	OTC-I	No dead skin cells	Visibility of dead skin cells	Non-toxic
		Wang et al. (2020)	CMCS	Fibroblasts grow well	NA	NA
		Gao et al. (2016)	CH and AgSD	70.14% cell survival	NA	NA
		Chen et al. (2020)	PVA and mGO	Noncytotoxic	NA	NA
		Ceylan et al. (2021)	PVA, CH, HP and propolis	Fibroblasts attached to the biomatrix	NA	NA
		Cui et al. (2021)	CH	Nontoxic	Cytotoxic	NA

### Cellular Response

Excellent biocompatibility is an expected feature for the promising scaffolds to be applied as medical-based materials, especially wound dressing. Furthermore, with GNP as a crosslinking agent, scaffold formulations have been reported to be non-toxic against human skin cells. Thus, the usage of GNP is highly recommended as a potential natural crosslinker for skin biomatrix fabrication.

Arif et al. (2020)and Karaki et al. (2016) have investigated the response of dermal fibroblasts and epidermal keratinocytes that were seeded onto the GNP-GEL sponges. The proliferation and cytotoxicity tests were done by using 3-(4,5-dimethylthiazol-2-yl)-2,5- diphenyltetrazolium bromide (MTT) and live/dead assay, respectively. The GNP-GEL scaffolds displayed superior biocompatibility than EDC-crosslinked. The data also demonstrated that both skin cells could proliferate within 7 days and no toxic effect of GNP-GEL sponges has been found. A colorimetric method was employed by Nair et al. (2020) to observe the proliferation of dermal fibroblasts on the COL films with the addition of EDC, GNP, and TG2 as crosslinkers. It was found that no significant proliferation was shown on the uncrosslinked films over the 7 days of observation. The increment of proliferation was observed on GNP-crosslinked films within 4 days of the experiment. Meanwhile, there was no significant difference in fibroblasts proliferation on the films that were treated by TG2 and EDC-NHS on day 4. These data suggested that GNP-crosslinked films were better than TG2- and EDC-NHS-crosslinked groups in terms of cells-scaffold interaction.

**FIGURE 7:**
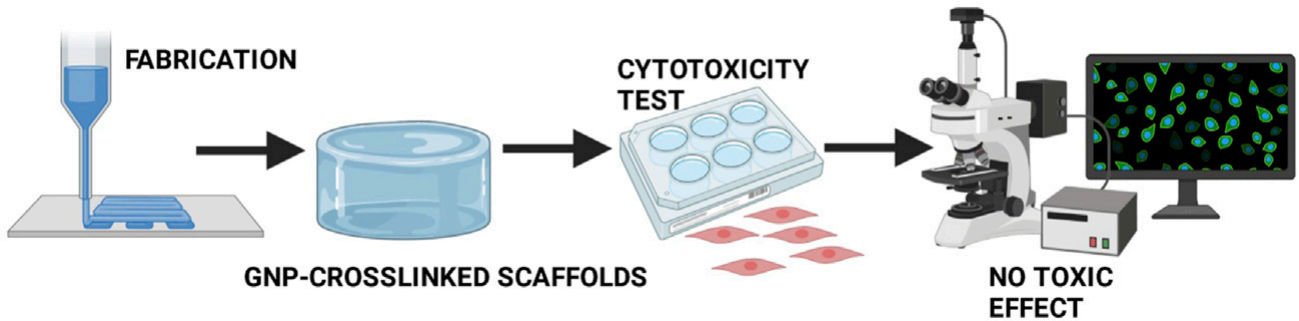
No toxicity of GNP-crosslinked scaffolds. Several studies have proven the survival of human skin cells in the culture during GNP-crosslinked intervention.

Furthermore, Wang et al. (2020) demonstrated that carboxymethylchitosan (CMCS) dressings (sponge, hydrogel, woven, and membrane), were treated with 10% of GNP solution, supported the proliferation effect of human skin fibroblasts for 2 weeks. Confocal laser scanning microscope (CLSM) photos prove that fibroblasts grew well and show spindle-shaped morphology on the surface of all dressings. According to Gao et al. (2016), the CH-silver sulfadiazine (AgSD) hydrogel, which was added with 1.65 mM GNP, exhibited 70.14% cell survival, and when the GNP concentration increased to 4.4 mM, cell viability could increase to almost 100%. *In vivo* evaluation and mice’s excision and burn cutaneous wound demonstrated that GNP-crosslinked AgSD hydrogel decreased the inflammatory cytokine and increased growth factors. This data represented that GNP-crosslinked AgSD hydrogel could enhance wound healing. Chen et al. (2020) have constructed films from a mixture of PVA, CH, and modified graphene oxide (mGO). The GNP solution (5 mg/ml) was then added to the mixture. The viability of HaCaT cells on the top of GNP-crosslinked. PVA-CH-mGO films were detected by cell counting kit-8 (CCK-8). The data exhibited no significant difference in cell viability within 5 days post-seeding, which confirmed the noncytotoxic effect of the films. Furthermore, (Fauzi et al., 2019; Fauzi et al., 2020) have observed the cytotoxicity of OTC-I sponges that were modified with GNP and carbodiimide (EDC) in 2019 and 2020. They found that GNP-crosslinked OTC-I did not kill the skin cells. Meanwhile, some of the skin cells were dead on the EDC-crosslinked OTC-I formulation. The latest investigations, that were done by Ceylan, 2021a; Ceylan, 2021b, have proven the survival of fibroblasts in the leaching medium of GNP-crosslinked-PVA-CH membranes that were loaded with *Hypericum perforatum* (HP) and propolis. Through SEM images, he also presented round spindle-like shape cells on the membrane which represented the attachment of fibroblasts. Cui et al. (2021) have published the nontoxic effect of GNP and the potential cytotoxicity of other crosslinkers, such as GTA and epichlorohydrin, in the fabrication of CH-based membranes for wound healing application. The aforementioned research confirmed the function of GNP in maintaining skin cell survival, as described in Figure 7.

Even though genipin is well-performed as a crosslinking agent in various natural polymers, it also possesses additional advantages, including the antiinflammatory and antioxidant effects, which contributed in expediting the wound healing process. Therefore, GNP intervention in bioscaffold fabrication could bring together dual or synergistic effects at a time. This unique characteristic might elevate the advancement in various explorations for medical products in the future. Thus, in this review, the effect of GNP on antiinflammatory and antioxidants has also been discussed for better understanding.

## Genipin Crosslinking Effect as Antiinflammatory and Antioxidant in Wound Healing

**FIGURE 8:**
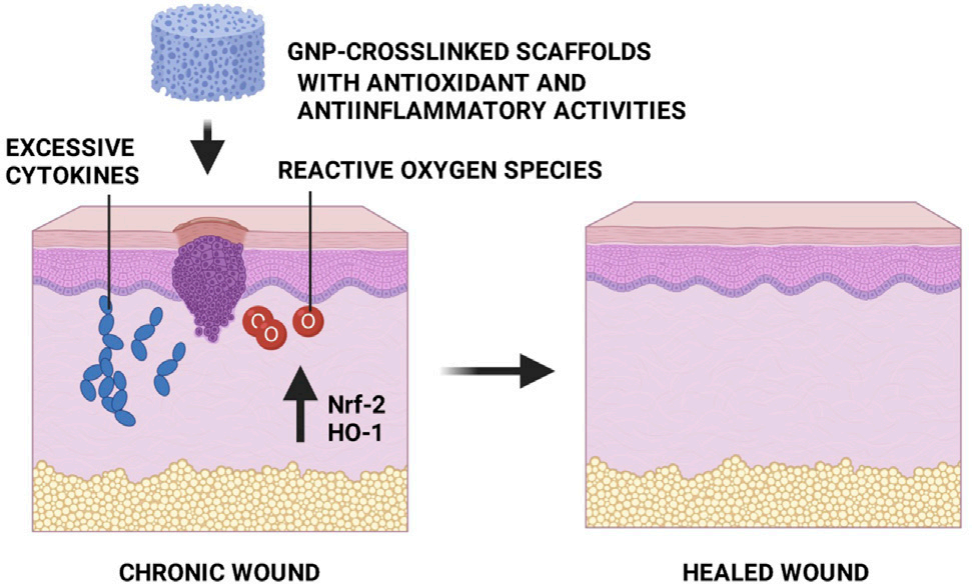
Effects of GNP in cytokine secretion. GNP decreased the production of cytokines which represented its antiinflammatory activity. This figure is reproduced from Szymanski et al. (2020).

Antiinflammatory and antioxidant activities of GNP have been well-established. Those properties are well-contributed in accelerating wound healing, especially difficult-to-heal wounds. It has been demonstrated that GNP reduced the inflammation in a murine macrophages cell line (RAW 264.7) and a murine microglial cell line (BV-2), which was stimulated by lipopolysaccharide (LPS) or interferon, through the inhibition of nitric oxide (NO), inducible NOS (iNOS), nuclear factor kappa-light-chain-enhancer of activated B cells (NF-κB), cyclooxygenase (COX)-2, interleukin-1β (IL-1β) and interleukin-6 (IL-6) (Aubert-Viard et al., 2019; Fauzi et al., 2020). Another study stated that GNP inhibited inflammatory response through blockage of inflammasome activation, which depends on the suppression of autophagy (Li et al., 2019a; Li et al., 2019b). Additionally, GNP was shown to IκB kinase (IKK) inhibition activity to alleviate inflammation. In addition, GNP could affect the PI3K/Akt signaling pathway and inhibit the expression of tumor necrosis factor-alpha (TNF-α). Furthermore, the antiinflammatory effect of GNP may be attributed to the inhibition of prostaglandin E-2 (PGE_2_) production through the blocking of COX-2 (Zhao et al., 2020; Meng et al., 2021). Szymanski et al. (2020) have reported that the presence of GNP in 3D human skin equivalent could suppress the secretion of other cytokines (Figure 8) such as interleukin-7 (IL-7), interleukin-8 (IL-8), interleukin-10 (IL-10), and interleukin-15 (IL-15).

**FIGURE 9:**
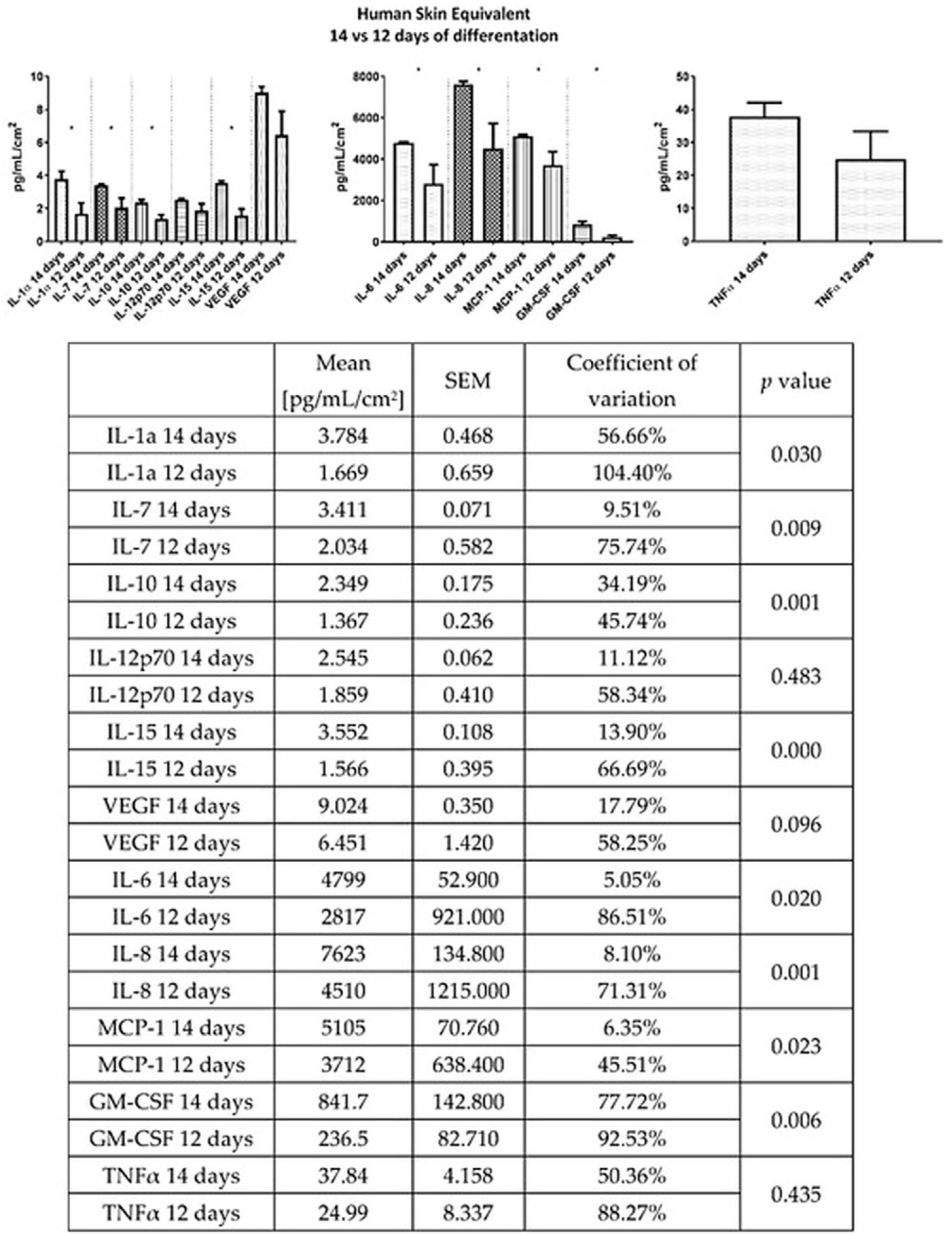
GNP-crosslinked scaffolds as an alternative treatment for skin wounds. Antiinflammatory and antioxidant properties from GNP will accelerate the healing process.

Another prominent study by Zhao et al. (2018) have reported that GNP possesses antioxidant activity in H_2_O_2_-induced epithelial cells. They suggested that the GNP treatment significantly increased the expression of heme oxygenase-1 (HO-1) and nuclear factor erythroid 2-related factor 2 (Nrf-2) in H_2_O_2_-induced epithelial cells. Moreover, the flow cytometry data exhibited that reactive oxygen species (ROS) levels and the number of apoptotic cells were decreased in H_2_O_2_-induced cells treated with GNP. Thus, Nrf-2 serves a significant role in regulating antioxidant enzymes, while HO-1 addresses a key role in protecting cells from oxidative damage. Another antioxidant action of GNP was evaluated by Zhao et al. (2020) whereby 50 mg/ml GNP reduced oxidative stress biomarkers, such as malondialdehyde (MDA) and myeloperoxidase (MPO) by 16.8% and 15.9%, respectively, 100 mg/ml GNP suppressed both biomarkers by 16.8% and 17.4%, respectively. Furthermore, it restored the antioxidant enzyme activities of catalase (CAT) and superoxide dismutase (SOD). Shin and Lee 2017 reported the potent cytoprotective effect of GNP against ROS-induced cytotoxicity through scavenging superoxide radicals and peroxynitrites in experimental models. In addition, they also wrote the role of GNP in decreasing mitochondrial ROS production. Altogether, the above findings suggested that GNP has a potential function in the wound closure process as an antiinflammation and antioxidant agent, which is beneficial for wound healing (Figure 9). In addition, it might be helpful as a therapeutic agent to be used in the production of topical treatment for skin wounds. The incorporation of GNP to any related biomaterials is a value-added key factor in advanced wound care management to expedite wound healing by synergistic effects, especially in difficult-to-heal wounds.

## Conclusion and Future Perspectives

In summary, crosslinking reaction is a method in skin tissue engineering to alter the characteristics of scaffolds for achieving desirable clinical output in skin wound healing. GNP is the most attractive plant-based crosslinking agent among the available crosslinkers due to its low toxicity and wound healing properties. In addition, the scaffolds manipulated with GNP offer porous structure, good swelling ability, excellent biocompatibility, and superior mechanical strength, which are crucial for wound care applications. Based on the aforementioned findings, GNP can be considered the most potential option in skin bioscaffold development. Furthermore, GNP is also a good choice in the future for several applications, such as cosmetics, pharmaceutics, and health supplements. Lastly, it is a promising candidate for topical treatment for inflammation in cream or gel. In addition, the biosafety and long-term stability study for biomatrix development should be explored more after crosslinking with GNP. These proposed parameters could provide a better understanding of its effectiveness and efficiency after prolonged storage. The possibility of using GNP in the future development of multifunctional smart biomaterials could expand the ability of GNP-incorporated biomatrix in preparing ready-to-use product design for the needful in personalized or precision medicine treatment strategy.

In terms of a clinical point of view, as of now, there is a limitation in stipulating or concluding the aforementioned characteristic of GNP-crosslinked biomaterials due to the lack or none of the published clinical trial findings worldwide. Thus, it may be difficult to tabulate the significance of utilizing GNP in clinical applications. However, this review has proven in several studies *via* preclinical model that GNP-integrated biomaterials’ effectiveness or performance provides excellent wound recovery. Prior to *in vivo* implantation, the selected biomaterials underwent tremendous physicochemical and mechanical evaluation by excellencing most of the previously mentioned properties. These findings could be an initial step or guidance for researchers worldwide to carefully select the best biomaterials for further evaluation in pilot or clinical trial studies. Again, the possible output for selected GNP-integrated biomaterials in the clinical study may provide different expectations or performances depending on multifactorial, including the type of wound, severity, patient’s health status, population, etc. At this point, a lot of clinical trial data is required to further evaluate the correlation between GNP-incorporated biomaterials development characterization with efficiency evaluation *via* preclinical model and clinical trial findings. Therefore, the accumulation of this beneficial information needs to be accomplished and achieved to contribute as a part of big data for future precision medicine in managing the wound care strategy.
